# Towards a TILLING platform for functional genomics in Piel de Sapo melons

**DOI:** 10.1186/1756-0500-4-289

**Published:** 2011-08-11

**Authors:** Mireia González, Meihong Xu, Cristina Esteras, Cristina Roig, Antonio J Monforte, Christelle Troadec, Marta Pujol, Fernando Nuez, Abdelhafid Bendahmane, Jordi Garcia-Mas, Belén Picó

**Affiliations:** 1 IRTA, Centre de Recerca en Agrigenòmica CSIC-IRTA-UAB, Carretera de Cabrils Km 2, 08348 Cabrils (Barcelona), Spain; 2 Institute for the Conservation and Breeding of Agricultural Biodiversity (COMAV-UPV), Universitat Politècnica de València, Camino de Vera s/n, 46022 Valencia, Spain; 3 Instituto de Biología Molecular y Celular de Plantas (IBMCP), Universitat Politècnica de València (UPV)-Consejo Superior de Investigaciones Científicas (CSIC), Ciudad Politécnica de la Innovación (CPI), Ed. 8E, C/Ingeniero Fausto Elio s/n, 46022 Valencia, Spain; 4 Unité de Recherche en Génomique Végétale (INRA-URGV), 2 rue Gaston Crémieux CP 5708, 91057 Evry Cedex, France; 5 Department of Plant Science, School of Agriculture and Biology, Shanghai Jiaotong University, 200030 Shanghai, People's Republic of China

**Keywords:** *Cucumis melo*, *inodorus*, TILLING, mutant, disease resistance, fruit quality

## Abstract

**Background:**

The availability of genetic and genomic resources for melon has increased significantly, but functional genomics resources are still limited for this crop. TILLING is a powerful reverse genetics approach that can be utilized to generate novel mutations in candidate genes. A TILLING resource is available for *cantalupensis *melons, but not for *inodorus *melons, the other main commercial group.

**Results:**

A new ethyl methanesulfonate-mutagenized (EMS) melon population was generated for the first time in an andromonoecious non-climacteric *inodorus *Piel de Sapo genetic background. Diverse mutant phenotypes in seedlings, vines and fruits were observed, some of which were of possible commercial interest. The population was first screened for mutations in three target genes involved in disease resistance and fruit quality (*Cm-PDS*, *Cm-eIF4E *and *Cm-eIFI(iso)4E*). The same genes were also tilled in the available monoecious and climacteric *cantalupensis *EMS melon population. The overall mutation density in this first Piel de Sapo TILLING platform was estimated to be 1 mutation/1.5 Mb by screening four additional genes (*Cm-ACO1*, *Cm-NOR*, *Cm-DET1 *and *Cm-DHS*). Thirty-three point mutations were found for the seven gene targets, six of which were predicted to have an impact on the function of the protein. The genotype/phenotype correlation was demonstrated for a loss-of-function mutation in the *Phytoene desaturase *gene, which is involved in carotenoid biosynthesis.

**Conclusions:**

The TILLING approach was successful at providing new mutations in the genetic background of Piel de Sapo in most of the analyzed genes, even in genes for which natural variation is extremely low. This new resource will facilitate reverse genetics studies in non-climacteric melons, contributing materially to future genomic and breeding studies.

## Background

Melon (*Cucumis melo *L.) is an important vegetable crop. Genetic and genomic information for this crop is increasing significantly due to several national and international projects [1]. A broad range of genomic tools are available today [2-7]. An effort is also in progress, through a Spanish initiative, to obtain the whole genome sequence of this crop [8]. These tools are generating a lot of information about genes involved in various biological processes, such as plant resistance and fruit quality [9,10]. However, the tools necessary for reverse genetic studies to conduct the functional validation of candidate genes in melons are still limited.

The TILLING (Targeting Induced Local Lesions in Genomes) method may represent an effective means of addressing limitations in melon research [11]. The application of TILLING has proved to be useful in identifying novel alleles in genes controlling traits of agronomic interest in legumes [12-15], cereals [16-23], solanaceous crops [24-26] and *Brassica *spp [27,28].

*C. melo *is a highly variable species divided into two subspecies, *melo *and *agrestis*. Most of the commercial cultivars belong to the *inodorus *and *cantalupensis *groups of the subspecies *melo *[29]. Cultivars of these two commercial groups are quite different in plant, flowering and fruit traits. *Cantalupensis *cultivars are early flowering, frequently monoecious, and produce climacteric fruits (aromatic, medium sugar, soft fleshed and with a short shelf life), whereas *inodorus *are late flowering, mostly andromonoecious, and non-climacteric, producing non-aromatic, high sugar, firm-fleshed fruits with a long shelf life, and which are preferred in certain markets. These differences make breeding strategies and objectives quite different for the two groups of melons.

A melon TILLING platform generated from a monoecious climacteric *cantalupensis *genotype has recently become available [30], and has proven to be useful for improving the shelf life of climacteric melons. A TILLING platform generated in an *inodorus *background might represent a useful resource for functional studies and for breeding non-climacteric melons.

In this paper, we present the development of an EMS-mutagenized population from an andromonoecious non-climacteric *inodorus *melon type (*Cucumis melo *L. subspecies *melo *cv Piel de Sapo). In total, 2,368 M2 families were obtained. A diversity of interesting mutant phenotypes were observed in the field. In addition, several mutant families have been identified in several genes that were selected based on their potential contribution to fruit quality and disease resistance.

## Methods

### EMS mutagenesis of melon seeds

The double haploid line, M62-113 (from Semillas Fito S.A.), belonging to the Piel de Sapo commercial type (*Cucumis melo *subsp *melo *var *inodorus*), was used as the starting cultivar. This is the parent of the melon genetic map and is one of the parents of the DHL line selected for the sequencing of the melon genome. Different batches of a total of ~12,000 M62-113 seeds were treated with the alkylant mutagen ethyl methanesulfonate (EMS). Different EMS concentrations were tested, and 1% was finally selected and tested on a larger batch of seeds. Seeds were first hydrated with tap water (16h), and were then soaked in tap water containing EMS concentrations (18 h). The treated seeds were thoroughly rinsed in tap water twice for 4 hours. After washing, seeds were placed on trays over wet paper, and were kept in a germination chamber overnight. ~5,000 mutant plants of this first (M1) generation were transplanted at the greenhouse. Plants were grown in two localities, in Barcelona (by Semillas Fito S.A. and IRTA) and in Valencia (by COMAV-UPV). Each M1 plant was selfed (1 fruit per plant) giving rise to the M2 seed.

### TILLING procedure

Twenty seeds were sown per M2 family, and the germination percentage was scored. Finally, a total of 2,368 mutagenized M2 families, which produced at least 10 viable seedlings in at least two plantings, were sampled for DNA extraction. 1-cm discs of tissue from 10 individual plants per M2 were pooled, and total DNA was extracted, quantified, diluted and organized into twenty-five 96-well plates. Using equivalent amounts of DNA from individual M2 families, the samples were pooled four-fold and organized into a 96-well format. Seeds of all pooled M2 populations are maintained at the Genebank of the COMAV-UPV, coded as F (S. Fito), I (IRTA) or C (COMAV-UPV) plus the corresponding number.

**Figure 1:**
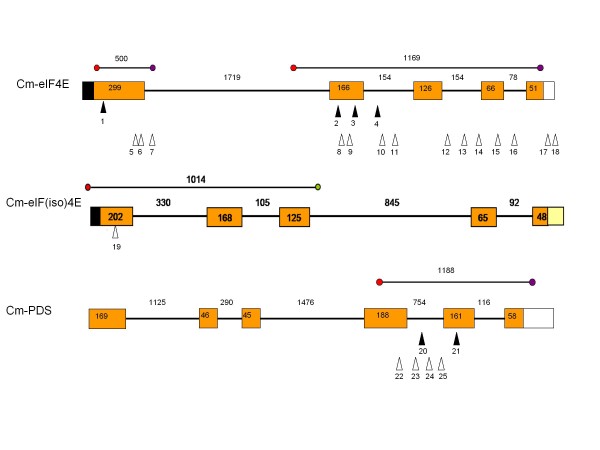
**Gene structure of the target genes screened in both populations,*inodorus *and *cantalupensis*, (eIF4E, eIF(iso)4E and PDS)**. Boxes represent exons and lines introns. White and black boxes indicate 3'UTR and 5'UTR regions. Amplicons analyzed by TILLING are indicated. Black and white triangles indicate mutations found in the *inodorus *Piel de Sapo and *cantalupensis *Charentais populations, respectively. Numbers correspond to the mutations described in table 3.

**Figure 2:**
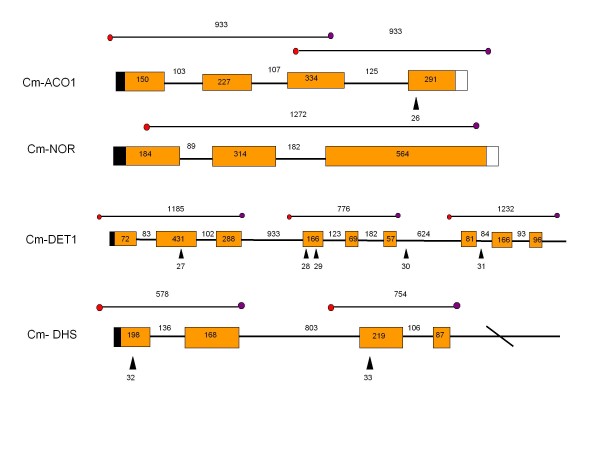
**Gene structure of the target genes screened in the *inodorus *population (ACO-1, NOR, DET-1 and DHS)**. Boxes represent exons and lines introns. White and black boxes indicate 3'UTR and 5'UTR regions. Amplicons analyzed by TILLING are indicated. Black triangles indicate mutations found in the Piel de Sapo population. Numbers correspond to the mutations described in table 3.

Primer sets were designed from 3 genes that were selected based on their potential contribution to fruit quality and disease resistance: *Cm-eIF4E *(translation initiation factor 4E) and *Cm-eIF(iso)4E *(translation initiation factor E, Isoform) which are involved in resistance to viruses [31], and *Cm-PDS *(Phytoene Desaturase), which is involved in carotenoid synthesis [32]. The CODDLe program (Codons Optimized to Deliver Deleterious Lesions) [33] was used to select the regions most likely to harbor deleterious changes induced by EMS (Table 1). These 3 genes were tilled in the Piel de Sapo population as well as in a set of 2,483 available lines of the *cantalupensis *TILLING population reported in [30]. Four additional genes involved in fruit ripening were tilled in the Piel de Sapo population: *Cm-ACO1 *(ACC oxidase 1), involved in the conversion of ACC into ethylene [34]; *Cm-NOR *(non-ripening), a transcription factor related to ethylene-sensitive/insensitive phenotypes [35]; *Cm-DET1 *(de-tiolated-1), a negative regulator of light-mediated responses that affects carotenoid and flavonoid pathways in tomato and other crops [36,37]; and *Cm-DHS *(Deoxyhypusine Synthase), mutations of which delay fruit softening in tomato [38]. Primer sets used for these additional genes are the same as those used in [30]. The amplicons analyzed for each gene are indicated in Figures 1 and 2.

**Table 1:** Primer pairs used to amplify the 3 target genes tilled in both EMS-mutant populations

Gene	Forward (*LabelledIRD700)	Reverse (*LabelledIRD800)
Cm-eIF4E 1^st ^amplicon	GACTCAAACGCCTAACAGAAAATC	CTATCTCACCAAGTTTCCTAAATT
	
	*GAGGGCGGTGCCATTCTTCTTCGG	*TCCCTAAATCGAACCAAGAAACGCC

Cm-eIF4E 2^nd ^amplicon	TTGTTTCGTGTTGACATGTCCATC	AGCATGCATTTCACCTCATTGGCT
	
	*TGCTTGGCTGTTAATTTATCTCTGC	*GTCAAGTACAGAACAAGAATCTGAG

Cm-eIF(iso)4E	GATCGATAATTTCCCTTTTC	TGACAGGCTTAAGCACTATG
	
	*ACCCCAATTCATTCTAGGG	*TGTGCAAGCGCAACAAGGTAC

Cm-PDS	CAATGGGATGCTTAGATCAAC	TCCTTCCAAAATTACCCTAG
	
	*GGATTTCTCGGAGTGACTCGG	*GCTCTCGAGCAACTAGCACT

Nested PCR was used in all cases with labeled primers in the second PCR.

Nested PCR was used to improve the specificity of amplification. The PCR reactions were performed in a 25 μl volume consisting of dH_2_O, 1× PCR buffer (Promega Corp, Madison, WI), 2.5 mM MgCl_2_, 0.2 mM dNTPs, 1 U Taq polymerase, 0.4 mM forward and reverse primers, unlabeled in the first reaction, and 700 nm and 800 nm 5' labeled (MWG Biotech AG, Germany) in the second reaction, and 10 ng DNA. The thermocycling conditions were 95°C for two minutes for initial denaturing, followed by 35 cycles of 95°C for 20 seconds, 63-69°C (specific for each primer pair) for one minute, 72°C from 30 seconds to one minute and one cycle of 72°C for five minutes. PCR products (~0.2 μl) were separated in 1% agarose gels. The PCR products were heated and cooled in a thermocycler (a gradient starting at 94°C and decreasing 0.1°C per second to 4°C) to form the heteroduplexes. Once the heteroduplexes were formed, the products were treated with Endo I. Digestions were performed in 30μl of final volume with dH2O, 3μl digestion buffer, 150 ng of PCR product and 3μl of EndoI (diluted 1/5000). The products were incubated at 45°C for 20 minutes to digest mismatches in the heteroduplex. Digestion was stopped by adding 5μl of 0.15 M EDTA and placing the reaction on ice. After the digestion was completed, the products were filtered through a Millipore MultiScreen filter plate that was packed with hydrated Sephadex G-50 medium beads (Amersham Biosciences). Samples were concentrated in the Speed Vac at 65°C for 45 minutes. Loading dye was added and the PCR products were denatured and loaded onto a polyacrylamide gel attached to a LI-COR 4300 DNA Analyzer (LICOR, Lincoln, NE) for separation. A total of 903 pools were assayed (592 four-fold pools of the Piel the Sapo population and 311 eight-fold pools of the *cantalupensis *population). Once a mutation was revealed in a pool, the corresponding families were analyzed individually to select the mutant M2 family. Mutation was confirmed by sequencing in the pooled DNA, in the individual M2 families and in several individual plants used in the segregation studies. The effect of the mutations was analyzed with SIFT (Sorting Intolerant from Tolerant, http://blocks.fhcrc.org/sift/SIFT.html) [39], which predicts whether an amino acid substitution affects protein function. Scores below 0.05 are predicted to affect protein function.

### Phenotyping of M2 and M3 individuals

Seedling phenotypes were systematically evaluated in the M2 generation. Additionally, in order to increase the number of seeds in our mutagenized population, we obtained the M3 generation. M2 populations that are being reproduced are also being inspected for phenotypes that are distinct from the wild type in plant, flowering and fruit traits. To date, about 800 M2 plants have been reproduced by S. Fito. M3 plants are also being analyzed for ethylene-response mutants by growing seedlings in darkness with and without an air flux containing 10 microL L-1 ethylene, and analyzing the "triple response".

## Results

### Generation of an EMS mutant population in the Piel de Sapo background

We selected 0.5%, 1 and 1.5% for generating a pilot population of ~600 lines, and studied the effect of EMS dosage on M2 seed viability and seedling vigor. Increased EMS concentration led to an increased percentage of M2 fruits in which seeds were non-viable or in which the number of viable seeds was too low to perform TILLING analysis (13.24%, 27.75% and 31.38%, respectively). Only a slight reduction in the average germination rate was observed in M2 families obtained from 0.5% M1 (93.51%), whereas higher reductions were obtained in 1% and 1.5% (82.73 and 75.94%, respectively). In addition, seedling growth was more restricted at the 1.5% concentrations. In contrast, the progeny from the 1%-treated M1 were robust. Based on these results, 1% EMS was used in the end to complete the mutagenized melon population. This EMS dose produces an acceptable level of M1 seed survival, fertility in M1 plants to set viable M2 seeds, and vigor in M2 seedlings. From a total of 3,140 M1 fruits finally obtained, 2,368 M2 families were sampled for DNA and used for TILLING purposes.

### Phenotyping of the mutagenized population

**Figure 3:**
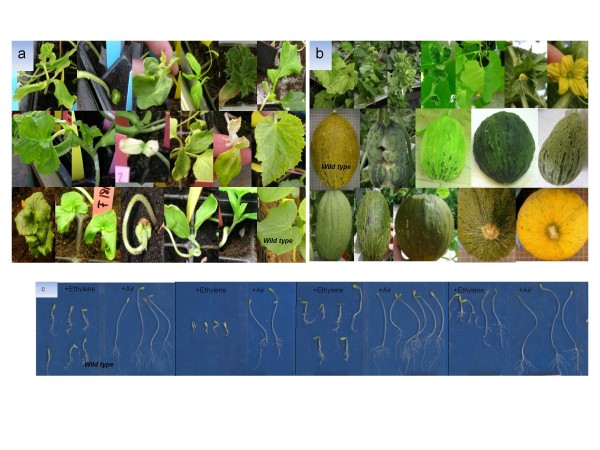
**Images of selected mutant phenotypes observed in the melon TILLING population**. a) Mutant seedlings found in M2. From left to right and from top to bottom, variations in cotyledons number and position, alterations in leaf and shoot morphology, albinism and chlorosis, including virus-like symptoms, dwarfing and altered gravitropism compared with seedling wild type. b) First row: Mutations found in adult M3 plants, chlorosis, dwarfing and leaf and flower deformation. Second row: alterations in fruits in comparison with wild type, deformation, chlorosis, rough surface, and pear-shape. Third row: fruit phenotypes of interest for melon breeding, variation in shape, longer and rounder melons, and melons with a subtle aroma and formation of abscission layer with or without color change. c) Variation in triple response: M2 families showing a more intense response to ethylene than wild type and others segregating for the response.

Ten plants per M2 line were scored for seedling phenotypes. A large number of the mutations affected cotyledons, with 5% of the M2 families showing variation in their number position and shape. About 4.3% of the M2 families segregated for "dwarf" or "semidwarf" plants. Albinism and chlorophyll deficiency occurred in 2.1% of the M2 families. Over 2% of the families showed alterations in leaves and shoot morphology. Gravitropic mutants and necrosis also appeared, but were less frequent. Figure 3 shows several seedling phenotypes. M3 families were characterized for plant flowering and fruit traits. Fruit traits of interest for melon breeding were identified: longer and rounder fruits than the standard Piel de Sapo fruits, with a lighter rind color, without the typical rind spots, and with different degrees of netting. Some lines have a subtle aroma and showed a color change and the formation of an abscission layer near maturity, traits that are more similar to climacteric *cantalupensis *types than to the standard non-climacteric *inodorus *Piel de Sapo. Differences in the dark-grown seedlings' response to ethylene were also observed. Wild type M62-113 showed the typical triple response: exaggeration of the apical hook, together with the inhibition of hypocotyl and root growth as well as the radial swelling of the shoot [40], whereas some M3 families showed responses of varying intensities (Figure 3).

### Identification of mutations in target genes by TILLING

The TILLING approach allowed us to identify nucleotide changes in two of the three gene targets first screened in the Piel de Sapo population (Table 2). EMS-induced changes were mostly G:C to A:T transitions (67%) (Table 3), as was expected due to the frequent alkylation of guanine residues by EMS, thus forcing mispairing with T [41]. Four mutations were exonic, most of which were determined to be missense mutations, only one being silent. A graph of the target genes marking the location of the induced polymorphisms is shown in Figures 1 and 2.

**Table 2:** Comparison of the mutations found by TILLING in the gene targets screened in the two EMS-mutagenized melon populations, Piel de Sapo (PS) and Charentais (CH)

	PS Population	CH population
Gene	Exon/Intron-UTR	

Cm-eIF4E	3/1	5/9

CmeIF(iso)4E	0/0	1/0

Cm-PDS	1/1	1/3

TOTAL	4/2	7/12

**Table 3:** Details of the mutations found by TILLING in the gene targets screened in the two EMS-mutagenized melon populations, Piel de Sapo (PS) and Charentais (CH)

Gene/population	Amplicon size (bp)	Identified mutants	N°^1^	Exon/Intron	Position	AA change	M2 Family	M2 segregation (WT/H/M)^2^	SIFT score
Cm-eIF4E/PS	500	1	1	Exon 1	A 74 G	R 25 K	F2036	9/7/8	0.00
	
	1169	3	2	Exon 2	G 385 A	E 129 K	F57	5/3/0	0.00
			
			3	Exon 2	G 465 A	T 155 T	F1880		
			
			4	Intron 2	G/C		F959		

Cm-eIF4E/CH	500	3	5	Exon 1	G 289 A	E 97 K	53		0.02
			
			6	Exon 1	C 294 T	F 98 F	2532		
			
			7	Intron 1	G/A		1420		
	
	1169	11	8	Exon 2	C 401 T	A 134 V	1229		0.10
			
			9	Exon 2	G 407 A	G 136 E	444		0.00
			
			10	Intron 2	C/A		1287		
			
			11	Intron 2	C/T		1115		
			
			12	Intron 3	G/A first nt of intron	splicing	1667		
			
			13	Intron 3	C/T		2646		
			
			14	Intron 3	C/T		1233		
			
			15	Exon 4	G 616 A	E 206 K	2060		0.09
			
			16	Intron 4	C/T		2035		
			
			17	3'UTR	T/C		1368		
			
			18	3'UTR	G/A		2163		
									
					222 TGA				

CmeIF(iso)4E/PS	1014	0	-						

CmeIF(iso)4E/CH	1014	1	19	Exon 1	G 128 A	R 43 K	M1587		1.00

Cm-PDS/PS	1188	2	20	Intron 1	C/T		F1151	4/7/4	
			
			21	Exon 2	C 560 T	S 227 F	C384	6/9/0	0.00

Cm-PDS/CH	1188	4	22	Exon 1	C 395 T	S 172 F	1182		0.01
			
			23	Intron 1	C/T		2495		
			
			24	Intron 1	T/C		1460		
			
			25	Intron 1	C/T		1226		

Cm-ACO1/PS	933	0	-						
	
	933	1	26	Exon 4	C 728 T	T 243 I	C142	10/6/3	1.00

Cm-NOR/PS	1272	0							

Cm-DET1/PS	1185	1	27	Exon 2	T 402 A	L 135 L	I76		
	
	776	2	28	Exon 4	G 760 A	V 255 I	F201	2/11/2	0.37
			
			29	Exon 4	C 848 T	S 284 L	F1876	2/4/3	0.25
	
	1232	2	30	Intron 6	G 2981 A		F1248		
			
			31	Intron 7	C 3211 T		C487		

Cm-DHS/PS	578	1	32	Exon 1	G 184 A	V 62 I	F 1728	13/20/0	0.58
	
	754	1	33	Exon 3	G 610 A	V 204 I	C348	2/6/0	0.27

^1^Number of mutations corresponds to those represented in Figures 1 and 2

^2 ^For some mutations, it was difficult to obtain homozygous MM plants as seedlings showed altered development.

Four new alleles were reported for *Cm-eIF4E*, defined by 4 mutations. Two (in exons 1 and 2) were predicted to affect protein function according to SIFT (p<0.05). The segregation of the missense mutations was analyzed in each mutant M2 family (Table 3) and homozygous individuals for each mutation are being produced for phenotypic analysis. Two new alleles were reported for *Cm-PDS*. Mutations were detected in the first intron and in the second exon of the amplicon (Figure 1). The exonic mutation was predicted to affect protein function.

**Figure 4:**
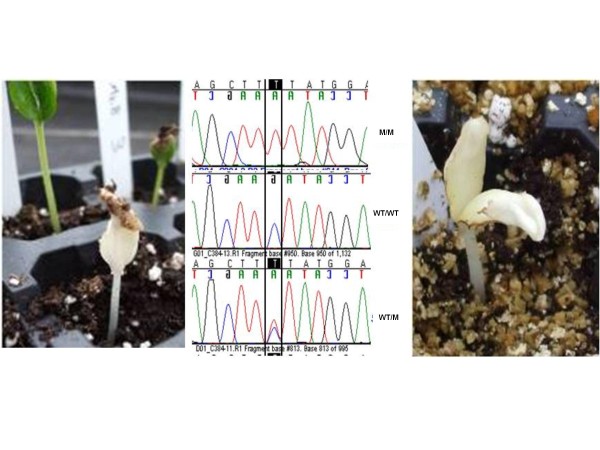
**Verification of genotype/phenotype relationship in the *Cm-PDS *gene**. Chromatograms of wild type (WT), homozygous mutants (M) and heterozygous are shown for the C 560 T transition altering codon 227 S (serine) to F (phenylalanine) and predicted to affect the protein function. Segregation of the mutation was confirmed in M3 seeds from a heterozygous M2 plant. The albino was the only plant homozygous for the mutant allele; the remaining M3 plants were wild type or heterozygous.

The genotype/phenotype segregation was verified for the *Cm-PDS *mutations in 15 plants of each M2. The intronic mutation found in M2 family F1151 (mutation 20 in Figure 1) presented a Mendelian segregation of 4 wild type/7 heterozygous/4 homozygous mutant (fitting to a 1:2:1 ratio) (Table 3). No associated phenotype was observed, which agrees with the position of the mutation in an intronic region. The M2 family C384 carried a missense mutation in exon 2 (mutation 21 in Figure 1). The segregation of M2 was 9 heterozygous/6 wild type. In this family, we observed that some plants showed delayed growth and even death at the cotyledon stage, and therefore could not be sampled. M3 seeds from a heterozygous M2 plant were used to repeat segregation analysis. Fourteen plants germinated out of the 20 seeds sown. Again, plants with delayed growth were observed, but we also found a seedling with albino cotyledons and hypocotyl (Figure 4). This seedling was carefully grown until tissue could be sampled for DNA extraction. The albino was the only plant homozygous for the mutant allele; the remaining M3 plants were wild type or heterozygous. This mutant line showed a similar albino phenotype comparable to the previously reported phenotypes for *PDS *disruption [32].

To further evaluate the mutation rate of the Piel de Sapo population, we extended the TILLING screen to 4 additional genes involved in fruit quality. Mutations were found for all genes, except for *Cm-NOR*, with *Cm-DET1 *being the most mutated gene. Six mutations were exonic, most determined to be missense, only one silent, but all were predicted to be tolerated (Figure 2, Table 3).

### Mutation efficiency

We used these results to calculate the mutation rate of the population. A total of 14 point mutations were detected out of the total 11,534 bp analyzed. Previous studies reported the difficulty in tracking mutations on the ends of the fragments (~100 bp) [42]. Therefore, 200 bp was subtracted off each amplicon, and we considered the length screened in this study to be 9,134 bp. The overall mutation density was then calculated by dividing the total base pairs screened, which includes the sum of the total length of the 12 amplicon sizes × the total number of individuals screened, by the total number of mutations revealed by TILLING ((9,134 × 2,368)/14 = 1,544,950). It was calculated to be ~1/1.5 Mb. The mutation frequency as determined by TILLING is coherent with the moderate level of phenotypic mutants found in our population.

In order to compare the *inodorus *and the *cantalupensis *populations, the same genes were screened in a set of 2,483 lines of the Charentais population [30]. The number of mutations was higher, and new alleles were found for the three genes (Table 2 Figure 1). The spectrum of observed changes was similar to that found in the Piel de Sapo population (Table 3). Only one mutation was found for *Cm-eiF(iso)4E*. *Cm-eIF4E *was also the most mutated gene with 14 mutations: seven intronic, two in the 3'-UTR, and five exonic. Of the exonic mutations, two, located in exons 1 and 2, were not tolerated (p<0.05). One intronic mutation was located in the first nucleotide of intron 3, and may affect a splicing site. TILLING analysis also revealed four mutations in *Cm-PDS*, one of which was not tolerated. The 19 point mutations discovered in the 3,871 bp analyzed in the Charentais population make a mutation rate of ~1/401 Kb ((3,071 × 2,483)/19 = 401,331). This is about 3 times higher than that found in the Piel de Sapo population. This high mutation rate in the *cantalupensis *population has also been observed by screening additional genes in the final version of the population, which consists of 4,023 lines [30].

## Discussion

The TILLING platform reported here is the first ever developed in an andromonoecious non-climacteric *inodorus *melon genetic background. The mutation rate in the Piel de Sapo population was moderate. It is less mutated than the *cantalupensis *population that was developed using a different genotype and a different protocol for treating the seeds, which resulted in increased seed viability at higher EMS doses (1 to 3%). Our attempt was the first with Piel de Sapo melons, and results suggest that our EMS treatment causes a high level of seed lethality. Regarding the protocol, Charentais seeds were not hydrated before being treated. Seed hydration has been reported to activate cellular division and induce the repair of DNA damage [43]. Differences in seed structure between genotypes, as well as an increase in the efficiency and accuracy of the repair of the induced lesions may explain the differences between populations. Perhaps, using a higher EMS dosage and/or an extended length of mutagen application, combined with a more intense wash length, and a more accurate seedling nursery would have increased mutation efficiency. Other authors that also found variable results in mutation frequency when using EMS within a crop suggest the use of alternative mutagens as a way for getting a higher density of mutations with less toxicity to the treated seeds [20].

**Figure 5:**
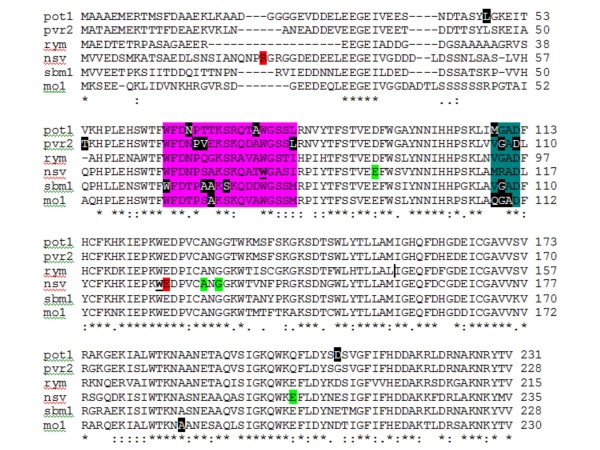
**Localization of eIF4E amino acids differing between virus-susceptible and virus-resistant genotypes of pepper (*Capsicum *spp., *pvr2*), tomato (*Lycopersicon *spp., *pot1)*, lettuce (*Lactuca *spp., *mo1*), pea (*Pisum sativum*, *sbm1*), melon (*Cucumis melo*, *nsv*) and barley (*Hordeum vulgare*, *rym*) in the predicted amino acid sequences (protein alignment of the susceptible genotypes)**. Black boxes indicate amino acids differing between susceptible and resistant genotypes. The two protein regions with clustering of amino acid substitutions are highlighted in pink and blue. In red are the mutations detected by TILLING in our PS population, in green, the mutations detected in the Charentais population. The conserved tryptophan residues reported to be required for cap-binding activity are underlined in the *Pisum *sequence [48].

Despite this higher mutation rate in the Charentais population, the efficiency of both populations in producing mutations predicted to be damaging to the proteins in *Cm-eIF4E *and *Cm-PDS *was similar. The new mutations found in *Cm-eIF4E *could be of interest, more so when this factor is highly conserved in eukaryotes. The natural diversity of *Cm-eIF4E *has been studied in melon and related species by EcoTILLING [31] and no variation has been reported within *Cucumis melo*, except for the *nsv *mutation (a point mutation of Leu228-His in exon 5), which controls resistance to *Melon necrotic spot virus *[44]. Variants in exons 1 to 3 were identified using EcoTILLING in another *Cucumis *species, *Cucumis zeyheri*, a wild relative of melon, isolated from the cultivated species by strong crossability barriers. Therefore, our new *Cm-eIF4E *alleles represent non-transgenic variants absent in nature. However it remains to be demonstrated if these mutations will lead to a gain of function, thereby producing new functional phenotypes, or to a loss of function. In other crops, recessive resistance to virus results from defective forms of eIF4E, for example in pepper (*pvr2*), pea (*sbm1*), tomato (*pot-1*), lettuce (*mo1*) and barley (*rym) *[45-47]. In most cases, resistance results from a few amino acid changes clustered in two neighboring regions of the *eIF4E *structure, located near the structural pocket involved in CAP-binding [48]. Some of the coding changes identified in our TILLING assay (in exons 1 and 2) are located near these two regions, close to the conserved tryptophan residues required for CAP-binding activity (Figure 5). The other coding mutations are found in different regions of the gene, but most affect highly conserved aminoacids. EcoTILLING has also shown that most natural variation in *Cm-ACO1 *occurs in exons 1, 2 and 3 [49]. No variants are found in large germplasm collections in exon 4. The effect of the identified mutation in exon 4 on ethylene production needs to be demonstrated.

The mutants found in *Cm-DET1 *and *Cm-DHS *are the first reported for these genes in *inodorus *melons. It has been demonstrated that phenotypes of the tomato mutants high pigment-2dg (hp-2dg) and hp-2j are caused by lesions in the *DET1 *gene. Point missense mutations and intron mutations directing alternative splicing have been reported in both the N-terminus and the C-terminus of the protein in *Arabidopsis *and tomato, suggesting that both ends of the protein are important for this function [50,51]. It has been reported that suppression of *DHS *delays loss of tissue integrity in senescing tomato fruit, leading to an extended shelf life. Further analysis will indicate if melon mutants *Cm-DET1 *and *Cm-DHS *have alterations in fruit senescence. Segregation studies are now being performed along with phenotype analysis of the mutant lines.

## Conclusions

The TILLING approach worked in *inodorus *melons as a way of identifying new heritable variants in candidate genes that are different from those present in natural populations. The cosegregation of a mutation predicted to alter the functionality of *PDS *with the albino phenotype expected for *PDS *disruption suggests that this is a promising approach for advancement in reverse melon genetics. It is also useful to analyze TILLING populations phenotypically, in order to use the phenotypes of interest readily in crop improvement. The novel fruit phenotypes could be of interest to diversify the market supply of Piel de Sapo melons. The information presented here will also be useful for creating new *inodorus *melon populations with a higher mutation rate. Completion of the melon genome sequence will provide many potential target genes of interest that may be functionally studied, facilitating future genomic and breeding studies.

## Competing interests

The authors declare that they have no competing interests.

## Authors' contributions

JG-M, MG and AJM performed the EMS mutagenesis. FN and BP contributed to the development and conservation of the M2 seed. BP, CR and CE extracted the DNA and performed the phenotypic analysis. DNA pooling, TILLING screens and analysis, and PDS mutant phenotypic analysis were done by MG, CT and MX. MX and MP participated in the sequencing of the eIF4E mutants in the M2 population. JG-M, AB, BP, and FN coordinated the study. BP, MG, CE, and JG-M were primarily responsible for drafting and revising the manuscript with contributions from co-authors. All authors read and approved the final manuscript.
